# Healthcare practitioners’ perspectives of providing palliative care to patients from culturally diverse backgrounds: a qualitative systematic review

**DOI:** 10.1186/s12904-023-01285-3

**Published:** 2023-11-17

**Authors:** Colette Burke, Owen Doody, Barbara Lloyd

**Affiliations:** 1 Milford Care Centre, Castletroy, Limerick, V94 H795 Ireland; 2 Department of Nursing and Midwifery, Faculty of Education and Health Sciences, University of Limerick, Limerick, V94 T9PX Ireland

**Keywords:** Palliative care, Systematic review, Cultural diversity

## Abstract

**Background:**

Palliative care practitioners are increasingly caring for patients and families from diverse cultural backgrounds. There is growing awareness of the influence of culture on many aspects of care in the palliative phase of an illness. However, disparities have been noted in the provision of palliative care to patients from culturally diverse backgrounds and challenges have been reported in meeting their needs and those of their families.

**Method:**

A qualitative systematic review of research papers identified through searching four databases. Papers were screened against inclusion criteria within the timeframe of January 2012 to March 2022. Data was extraction from all included studies and methodological quality assessed utilising the Critical Appraisal Skills Programme Tool. Thematic analysis followed Braun and Clarke’s framework and the review is reported in line with PRISMA guidelines.

**Findings:**

The search yielded 1954 results of which 26 were included for appraisal and review. Four themes were identified: communication and connection, the role of the family in death and dying, the role of education in addressing uncertainty, and institutional and societal factors. The findings highlighted challenges of communication and a fear of acting in a culturally insensitive way, the pivotal role of the family, the need for an individualised approach to care, the universality of needs when approaching end of life and the need for education of practitioners.

**Conclusion:**

These findings suggest that healthcare practitioners draw on their existing skills to adapt their practice to meet the needs of patients from culturally diverse backgrounds. However, results also indicate a need for further education and identification of educational approaches best suited to supporting healthcare professionals in practice.

**Supplementary Information:**

The online version contains supplementary material available at 10.1186/s12904-023-01285-3.

## Introduction

The care provided to the dying should reflect the kind of society we have created and live in [1] and the philosophy of Cecily Saunders (1918–2005), that care and treatment of the ‘whole person’ is best facilitated by a team, is a cornerstone of modern palliative care throughout the world [2]. Culture is an integrated pattern of human behaviour that includes language, thoughts, actions, customs, beliefs, and institutions of racial, ethnic, and social or religious groups [3]. Culture incorporates learned, shared knowledge of values, beliefs, and lifeways, and has rules that influence attitudes and behaviours that can be overt or covert [4]. Thus, culture is multifaceted, fluid and complex creating the potential to impact significantly on both health and illness [5].

The number of persons currently residing outside their country of origin has never been greater and numbers of migrants have increased progressively over the past thirty years [6]. According to the International Organization for Migration in 2019, Europe hosted 82 million international migrants, North America 59 million and Northern Africa and Western Asia (combined) 49 million [7]. Migration patterns are a constantly evolving phenomenon and occur for a variety of reasons such as work opportunities, education, lifestyle factors and conflict/war. The World Health Organization (WHO) highlights that healthcare for migrant and refugee populations should be provided in a gender-sensitive, culturally, and linguistically appropriate manner without stigma [8]. Disparities in the care experiences of culturally diverse patients exist [9] identifying a lack of knowledge of services [10], inadequate exchanges of information [11, 12], difficulties with the use of interpreters [9], difficulties explaining feelings and emotions [13], issues around disclosure of serious illness [14] and a focus on cultural background rather than the individuals experience [15].

If culture is the lens through which we view the world [16], then it must be acknowledged that the culture of healthcare practitioners, healthcare organisations and wider society will also impact interactions with culturally diverse patients and their families. Providing healthcare to patients from culturally diverse backgrounds has become a more frequent occurrence in developed countries [17] and a layer of complexity can be encountered when culturally diverse patients interact with healthcare in general and that this is particularly noteworthy in countries where cultural diversity is a more recent development [9]. Difficulties forming therapeutic relationships and the stress that this caused healthcare practitioners [18] and the need for practitioners to be aware of their own cultural frame of reference and to be able to recognize their own bias is evident [19]. Cultural competence in healthcare practitioners has been offered as a means of tackling the disparities in care provided to culturally diverse patients and to addressing the concerns identified by healthcare practitioners [20]. Within palliative care, it is acknowledged that the role of culture is significant [21] as it shapes how serious illness is understood, how suffering is articulated and how grief manifests in patients and their families and in the healthcare practitioners whom they encounter [22].

Culturally diverse patients are underrepresented amongst service users [23] and do not represent a homogenous group [24]. It is recognised that within the palliative phase of illness the vulnerabilities of patients and families are particularly evident [25]. All healthcare professionals governing bodies highlight that patients’ cultural background and ethnicity have an important effect on their health outcomes and healthcare professionals should try to understand culture and respond to individual needs. Therefore, the requirement for practitioners to provide care that is sensitive to the cultural background of patients is identified, reinforced, and heightened in palliative care as failure to address such needs cannot often be redressed later [26]. Thus, examining the experiences of healthcare practitioners providing palliative care to persons from culturally diverse backgrounds is important and this paper explores these experiences through a qualitative systematic review. While van Eechoud et al. [27] examined the experiences of oncology healthcare workers caring for ethnic minority patients the authors are unaware of any review from a palliative care context.

## Methods

**Fig. 1:**
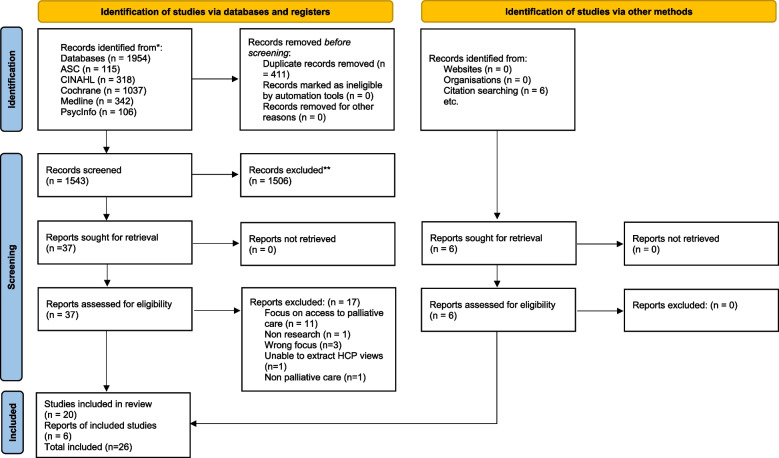
PRISMA 2020 flow diagram

This qualitative systematic review aimed to explore the perspectives of healthcare practitioners providing palliative care to patients from culturally diverse backgrounds. Wakefield’s guide to searching and reviewing literature [28] guided and supported and the review is reported as per the PRISMA checklist (Supplementary file 1) and PRISMA flowchart [29] (Fig. 1).

### Search sources and strategies

Searches were conducted within five electronic databases: PsycINFO, CINAHL, Academic Search Complete, Medline and Cochrane Library. The search strategy was based on the PICo framework (Population [healthcare practitioners], Phenomenon of Interest [perspectives] and Context [patients from culturally diverse background receiving palliative care]). The following terms were included in the search string strategy; first, for population ‘healthcare practitioners’, for phenomenon of interest ‘perspectives’ and for context ‘palliative care’ ‘culturally diverse’. All terms were joined utilizing the Boolean operators (“OR” within each search string and “AND” to combine - Table 1).

**Table 1:** Search terms used in database searches

Population	Healthcare practitioners	nurse OR nurses OR nursing OR doctor OR doctors OR physician OR physicians OR clinicians OR allied health personnel OR allied health practitioners OR allied health professionals OR healthcare providers OR healthcare personnel OR healthcare professionals OR healthcare workers OR healthcare staff
Phenomenon of interest	Perspectives	perspectives OR knowledge OR views OR attitudes OR experience OR experiences OR opinion OR opinions OR perceptions
Context Concept	Palliative care Culturally diverse	palliative OR hospice OR end of life OR dying OR terminal cultural diversity OR cultural minority OR minority groups OR minorities OR ethnic groups OR ethnicity OR migrant OR migrants OR immigrant OR immigrants OR indigenous OR cross cultural

Criteria for considering studies in this review were set as follows:

#### Inclusion criteria

- Primary research published in peer-reviewed journals.
- Papers written in English or translation available.
- Papers published between 01 January 2012 and 01 March 2022.
- Papers referring to the adult population over the age of 18 years.
- Studies which contain each of three elements: palliative intent to care, healthcare practitioners’ views or experiences and patients with culturally diverse background.
- Qualitative research design.

#### Exclusion criteria

- Secondary research – e.g., discussions, editorials, opinion papers, conference proceedings.
- Literature reviews - e.g., systematic, scoping, rapid, narrative, expert, integrative.
- Studies which focus primarily on advance directives and enrolment or access to hospice programmes.
- Non-qualitative methodologies.
- Studies where it is not possible to extract the views of the healthcare practitioner.

### Study selection and data extraction

All search results were exported to Rayyan intelligent systematic review software and duplicates deleted. The remaining titles and abstracts were screened against the inclusion criteria led by the first author (CB) during which the team met to discuss the screening criteria and 20% were doubled screening by the author and the team. All studies remaining were retrieved for full text screening where the authors worked in pairs to reach a final verdict. Reasons for exclusion were noted and reported (Fig. 1). A total of 1954 papers were identified with 411 duplicates leaving 1543 for screening. Title and abstract screening resulted in 1506 been excluded, leaving 37 studies for full-text screening where full-text were read and 17 excluded. Resulting in 20 papers meeting the inclusion criteria for this review. 6 additional papers were found through backward chaining from reference lists of included papers. Data extracted was performed on all 26 papers by the first author (CB) and reviewed by the other authors (BL/OD). The data extraction table (Table 2) developed reports: authors, year, title, country, primary aim, methodology, data collection, sample size, key findings and quality appraisal score [30].

**Table 2:** Data extraction table

Author, year, title, country	Primary aim	Methodology, data collection, participants, and sites	Key findings/comments	Quality appraisal,
Bellamy and Gott, (2013) [32] What are the priorities for developing culturally appropriate palliative and end-of -life care for older people? The views of healthcare staff working in New Zealand. New Zealand.	To explore the views and experiences of care staff regarding the provision of palliative and end-of-life care for Maori, Pacific Island and Asian, particularly Chinese, elders	Qualitative approach, based on grounded theory. 10 focus groups, 2 joint interviews. 14 doctors, 49 nurses, 17 other health professionals. Specialist palliative care teams, hospice, and community. Residential aged care facilities. GP practices. Hospital based teams.	Home preferred place of care, preference for family carers. Need for support/education of family to facilitate such care. Importance of institutional settings being able to accommodate large family groups Communication challenges: language and disclosure preferences, individual vs. collective decision-making Acknowledgement of heterogeneity within groups and importance of individual assessment.	8/10 80%
Debesay et al., (2014) [33] Facing diversity under institutional constraints: challenging situations for community nurses when providing care to ethnic minority patients. Norway.	To understand the challenges encountered by community nurses while providing home health care to ethnic minority patients in Norway.	Qualitative – based on a hermeneutics approach. In-depth interviews. 19 community nurses Home health care nursing teams.	Uncertainty/fear of causing offence when providing personal care. Discomfort/uneasiness caused by lack of familiarity with patients’ cultural norms. Challenges posed by perceived reluctance to talk about dying, nurses concerned especially when young children involved. Conflict between fulfilling own clinical role and respecting wishes of patient and/or family. Lack of institutional support in adapting services/supports to meet needs of minority patients, little time, lack of support for staff to develop expertise/share knowledge.	9/10 90%
de Graaff et al., (2012a) [34] Talking in triads: communication with Turkish and Moroccan immigrants in the palliative phase of cancer. The Netherlands.	To gain insight into the factors that influence communication between health professionals and Turkish and Moroccan immigrants in the palliative phase of cancer	Qualitative. Semi-structured in-depth interviews. 33 cases of patients with incurable cancer of Moroccan or Turkish descent. 6 patients, 30 relatives, 47 care providers. Various community sites and hospitals.	Multilingual triads featuring care provider, patient and relative. Little use of professional interpreters based on patient and family preference and perception that engaging interpreters is time-consuming. Concerns and differing opinions over what information is conveyed to patient by family. Difficulty determining wishes of patient and those of family members. Some subjects not broached by family or care providers due to communication challenges e.g., financial/family pressures, clarity on cessation of curative treatment. Varying response to conflict by care providers, some blamed family, some adjusted own communication strategies to align with patients/family and provided extra time, some acknowledged differences but proceeded as per own opinions and some sought middle ground in conjunction with family. Some communication scenarios similar to native Dutch family groups at end of life, however, care providers voiced they were more familiar with these situations so more comfortable dealing with them.	9/10 90%
de Graaff et al., (2012b) [35] Understanding and improving communication and decision-making in palliative care for Turkish and Moroccan immigrants; a multi perspective study. The Netherlands	To explore how communication and decision-making in palliative care amongst Turkish and Moroccan patients are influenced by different styles of care management between Turkish and Moroccan families and Dutch professional care providers	Qualitative. Semi-structured in-depth interviews. 33 cases of patients with incurable cancer of Moroccan or Turkish descent. 6 patients, 30 relatives, 47 care providers. Various community sites and hospitals.	Concept of ‘care management group’ used as key analytic tool to explore communication and decision-making. Quality of life emphasised by care professionals’ pursuit of cure emphasised by families. Role of family in ‘care management group’ not always adequately recognised. Communication and decision-making hampered by issues within and between patient and family members and members of the care provider teams. Some communication difficulties within family and within care teams not culture related but common to many groups. Some examples of communication between all parties which prove satisfactory to all.	9/10 90%
de Voogd et al., (2021) [36] Health care staff’s strategies to preserve dignity of migrant patients in the palliative phase and their families: A qualitative study. The Netherlands.	To gain insight into registered nurses’ and care assistants, difficulties, and strategies for preserving dignity in the last phase of life and their families.	Qualitative approach. 5 face to face focus groups with care staff. 1focus group with key informants. 29 care staff, 18 care assistants, 7 nurses, 2 social workers, 2 team leaders. 6 key figures with cultural mediation functions. Various Dutch nursing homes, 1 Dutch hospital.	Communication challenges with family involved differing opinion around decision-making, perceived family misconceptions about care or disease. Language barriers made eliciting patient preferences difficult and hampered development of relationships. Little use of interpreters in nursing homes settings. Ineffective/harmful strategies identified as eliciting patient’s wishes in private, attempting to change perspectives. Adapted language and explored religious/cultural matters with families. Some challenges also common to non-migrant Dutch population. Key respondents note use of religion/culture as explanation by migrant patients or families may mask other issues.	8/10 80%
Eckemoff et al., (2018) [37] End of life care for older Russian immigrants-perspectives of Russian immigrants and hospice staff, United States.	To gain insight into the views of older Russian immigrants, the adult children of older Russian immigrants and hospice care providers on end-of-life care.	Qualitative approach. Individual in-depth interviews. 4 Russian seniors, 5 Russian adult children, 4 hospice staff, 1 facility director, 1 nurse, 2 social workers.	Care for diverse patients seen as challenging. Training provided yearly to staff. Preference amongst older patients for care at home. Differing opinions between patients/families and staff on patient autonomy and decision-making. Differing views on use of non-family interpreters. Lack of understanding and information amongst patients and families regarding health services.	7/10 70%
Green et al., (2018) [38] ‘Death is difficult in any language’:a qualitative study of palliative care professionals’ experiences when providing end-of -life care to patients from culturally and linguistically diverse backgrounds, Australia	To elicit the experiences of palliative care health professionals when providing care for patients from CALD backgrounds which differ from mainstream Australian language and culture.	Emergent qualitative study, informed by grounded theory. 4 focus groups, 28 staff, 2 groups palliative care ward nursing staff. 1 group multidisciplinary community palliative care team. 1 group allied health team palliative care ward, 1 specialist care unit, 1 community palliative care service based at a hospital.	Death is difficult in any language. Need for individual assessment of communication preferences and needs of patient and family. Language barriers can impede communication. Use of interpreters infrequent, use of family interpreters challenging at times. Recognition of broader social and cultural factors which may influence care e.g., migration history, trauma, discrimination. Negotiation involved in addressing patient care needs with family involvement. Experience both challenging and satisfying for staff members.	10/10 100%
Henry and Timmins, (2016) [39] An exploration of specialised palliative care nurses’ experiences of providing care to hospice inpatients from minority ethnic groups- implications for religious and spiritual care. Ireland.	To gain an understanding of nurse’ experience of providing care to patients from minority ethnic groups within the specialist palliative care inpatient unit of an Irish hospice.	Qualitative hermeneutic phenomenology. Unstructured interviews. 5 nurses, each interviewed twice. 1 palliative care in-patient unit.	Facilitation of rituals of death and dying important part of nurse’s role. Lack of experience/familiarity source of uncertainty. Need to manage impact of large families on other patients. Conflict between clinical priorities e.g., analgesia and family beliefs. Importance of ‘proceeding slowly’ with acceptance of differing beliefs. Nurses perceived need for further education.	9/10 90%
Johnstone et al., (2016a) [40] Nursing roles and strategies in end-of-life decision making concerning elderly immigrants admitted to acute care hospitals. Australia	To gain insight into nurses understanding of culturally responsive end-of-life care, decision-making and quality of death for older immigrants.	Qualitative exploratory descriptive study. In-depth, semi-structured interviews, face-to-face or telephone. 22 registered nurses. 4 acute hospitals, medical-surgical, palliative care, aged care.	Initially nurses felt ill prepared, learnt ‘on the job’, from experienced colleagues. Lack of formal education, self-educated, an on-going process. Important role of nurses in assisting patient and family to face death. Recognition of heterogeneity within groups. Language barriers an impediment to communication. Important role of family in striving towards a good death, importance of building relationships. Importance of nurses’ role as advocate for patient and family.	9/10 90%
Johnstone et al., (2016b) [41] Assuaging death anxiety in older oversees-born Australians of culturally and linguistically diverse backgrounds hospitalised for end-of-life care. Australia	To describe and report the strategies nurses use to identify and help assuage the terror of death and related anxieties perceived in older immigrant patients and their families.	Qualitative exploratory descriptive study. In-depth, semi-structured interviews. Face –to-face: 13, telephone: 9. 22 registered nurses. 4 acute hospitals, medical-surgical, palliative care, aged care.	Participants identified components to death anxiety: fear of the unknown, fear of not having lived a meaningful life, fear of leaving behind loved ones. Importance of presence, ongoing explanations, and recognition of cultural influences, such as collective decision making, on family responses. Findings emphasised cultural similarities rather than differences in fear of death.	9/10 90%
Johnstone et al., (2016c) [42] Nursing strategies for engaging families of older immigrants hospitalized for end-of-life care: An Australian study. Australia.	To explore and describe the strategies nurses use to facilitate engagement with families of older immigrant patients from non-English-speaking backgrounds (NESB) hospitalised for EOL care.	Qualitative descriptive study. In-depth, semi-structured interviews. 22 registered nurses. 4 acute hospitals, medical-surgical, palliative care, aged care.	Nurses believed they had an essential role in engaging the families of older NESB families. Strategies used common to quality professional-family relationships irrespective of cultural background. Nurses recognised that challenging behaviour was often stress related and actively strove to ensure families were not labelled. Recognition that migration factors could influence interaction e.g., intergenerational difference/conflicting beliefs. Nurses recognised and emphasised the importance of including family members in care. Nurses demonstrated a ‘will to engage’ with families, also demonstrated willingness to engage with cultural difference. ‘Right attitude’ needed in addition to knowledge and skill to build and sustain good relationships between nurses and families.	6/10 60%
Johnstone et al., (2018) [43] Fostering trusting relationships with older immigrants hospitalised for end-of-life care. Australia.	To explore and describe the specific processes that nurses use to foster trust and overcome possible mistrust when caring for older immigrants of non-English speaking backgrounds hospitalised for end-of-life care.	Qualitative exploratory descriptive study. In-depth, semi-structured interviews. 22 registered nurses. 4 acute hospitals, medical-surgical, palliative care, aged care.	Recognition by nurses of importance of trust in all cultures. With older non-English speaking backgrounds (NESB) patients, particular importance of trust with potential differing attitudes to analgesia and conversations about diagnosis, and prognosis, death and dying was recognised. Nurses’ commitment to fostering trust demonstrated by care strategies that were ‘intentional, conscious, and conscientious. Strategies applicable to all patients at end-of-life (EOL).	9/10 90%
Khosla et al., (2016) [44] Perspectives of health care providers on US South Asians attitudes towards pain management at end of life. United States	To explore the attitudes and influences of US South Asians to pain management at end-of-life.	Multi-method qualitative descriptive study. 4 focus groups, 35 participants, 23 individual interviews. 57 participants -nurses -physicians - physician assistant -nurse administrators -chaplains -medical social workers. 1 large university-owned health system, 1 large non-profit healthcare organisations, community-based hospices.	General reluctance to take pain medication, participants caution against stereotyping but agree high prevalence. Some factors related to healthcare practices and beliefs in country-of-origin e.g., minimalistic attitude towards medication in general, limited access to pain medications in countries of origin. Strength of spiritual beliefs may influence decisions. Many patient and family concerns about medication (addiction, side effects, stoicism) very similar to those of general US population.	9/10 90%
Khosla et al., (2017) [45] Communication challenges and strategies of US health professionals caring for seriously ill South Asian patients and their families. USA	To explore the challenges faced by healthcare providers caring for seriously ill South Asians and their families. To explore strategies to address these challenges.	Multi-method qualitative descriptive study. 4 focus groups, 35 participants, 23 individual interviews. 57 participants -nurses -physicians - physician assistant -nurse administrators -chaplains -medical social workers. 1 large university-owned health system, 1 large non-profit healthcare organisations, community-based hospices.	Challenges identified- language barriers, HCP preference for professional interpreters sometimes resisted by families, difficulties with information modification when family interpret, telephone interpreters missing non-verbal cues. Difficulty identifying spokesperson in large family groups. Family wish to withhold information from patient caused disquiet among HCP. Passive communication style i.e., reluctance to explicitly state wishes identified among some patients and families. Some HCP’s felt South Asians culturally inexperienced in direct communication. Preference among some patients/families for specific gender of HCP identified. Potential benefit from use of cultural mediators identified.	7/10 70%
Khosla et al., (2019) [46] Health-care providers’ perspectives on decision-making among seriously ill patients on South Asian origin in the United States. USA	To better understand health-care providers’ perspectives on decision-making among seriously ill persons of South Asian origin.	Multi-method qualitative descriptive study. 4 focus groups, 35 participants, 23 individual interviews. 57 participants -nurses -physicians - physician assistant -nurse administrators -chaplains -medical social workers. 1 large university-owned health system, 1 large non-profit healthcare organisations, community-based hospices.	Acculturation and higher education levels resulted in increases participation in decision making by patients. Preference for physician- directed decision-making amongst some patients and families. Spiritual beliefs may be strongly influential on decisions re analgesia etc., involvement of spiritual leaders may be helpful. Family decision-making is a cultural norm, difficulties clarifying patient wishes, challenges for providers balancing US norms. Distribution of decision-making responsibilities can be complex- spokesperson may not always be decision-maker. South Asian patients and families may lack information or hold negative views of palliative options like other immigrant groups.	8/10 80%
Mahilall, R, and Swartz, L. (2021) [47] Spiritual care practices in the Western Cape, South Africa: the challenge of diversity. South Africa	To understand what spiritual care practices, exist in hospices in the Western cape, against the backdrop of multifaceted diversities.	Qualitative study. 2 focus groups. 23 participants- nurses, social workers, spiritual care workers, managers. 11 hospices.	Need for staff to have broad knowledge of differing religions, emphasis on non-judgemental approach, sensitivity and self-awareness. Respect for traditional spiritual practices and bereavement rites important. Awareness that patient’s spiritual needs may not align with patients’ own cultural or religious background. Team approach important. Resources- time, adequate staff.	6/10 60%
Milberg et al., (2016) [48] Health care professionals’ understandings of cross-cultural interaction in end-of- life care: a focus group study. Sweden	To explore end -of-life care providers’ understandings of patients with a migrant background.	Qualitative study. Focus groups. 60 MDT members, nursing, nursing assistants, medical, occupational therapy. 11 care settings, medicine/surgery/geriatrics/ specialised palliative home care/primary care.	Despite limited experience, anticipated cross-cultural interaction would be challenging. Feelings of uncertainty and stress when dealing with unfamiliar situations. Concerns about facing ethical dilemmas, language/communication difficulties with both patients and families. Expecting misunderstandings/worrying about unmet needs. Perceived lack of knowledge about other cultures.	8/10 80%
Neiman, (2019) [49] Nurses’ perceptions of basic palliative care in the Hmong population. USA	To describe basic palliative care from the acute nurses’ perspective with consideration for culturally diverse populations.	Qualitative theory generating study. 7 focus groups, 8 individual semi-structured interviews. 34 registered nurses. 3 acute hospitals, non-ICU/ED settings.	Importance in accommodating rituals/ accommodating cultural expression to ensure relationship with palliative care service is maintained. Importance of family involvement. Managing language barriers, concerns re quality of interpreter services and incomplete understanding/ reluctance of family to seek clarification. Perceived lack of specific cultural knowledge.	8/10 80%
Nielsen et al., (2013) [50] Maintaining distance from necessary intrusion: A postcolonial perspective on dying at home for Chinese immigrants in Toronto, Canada. Canada	To describe and examine how meanings of home condition negotiations of care for first generation Chinese immigrants with advanced cancer receiving palliative home care, family caregivers and home care providers.	Qualitative focused ethnographic study. In-depth individual interviews. Observation during home visits. 4 patients, 4 family caregivers. 3 home visiting nurses, 11 key informants. Community homecare organisations, palliative care centre.	Specialised knowledge of care providers resulted in subtle power imbalance. Nurses viewed education and preparation for dying as part of their role. Issues such as precarious employment of family members and lack of knowledge of services/benefits available reported. Dying at home involves complex emotional and physical adjustments and findings not ethno-specific. Need for health care providers to consider own cultural assumptions.	10/10 100%
Nielsen et al., (2015) [51] Patient-cantered care or cultural competence: negotiating palliative care at home for Chinese Canadian immigrants. Canada.	To describe and examine how meanings of home condition negotiations of care for first generation Chinese immigrants with advanced cancer receiving palliative home care, family caregivers and home care providers.	Qualitative focused ethnographic study. In-depth individual interviews. Observation during home visits. 4 patients, 4 family caregivers. 3 home visiting nurses, 11 key informants. Community homecare organisations, palliative care centre.	Perceptions of death as a taboo topic vary amongst patients and families. Perceptions of death as a taboo topic vary amongst healthcare providers. Possible cultural taboos such as not wanting to die at home found to have other explanations. The shared experience of dying supersedes culture, race, and religion. Language barriers an additional complication. Attempts to ‘match’ staff with patients based on language or culture needs to be carefully considered.	5/10 50%
Roider-Schur et al., (2019) [52] Migrate your mind: the role of palliative care in palliative cancer treatment. Austria	To gain insights into practical aspects of palliative care in the clinical encounter with terminally ill cancer patients with migrant backgrounds and their relatives.	Qualitative study. Semi-structured interviews. 21 MDT members, medical, nursing, social work, psychology, spiritual care. 1 site providing cancer care, OPD, oncology day ward, oncology ward, palliative care ward and radiation oncology.	Identified means by which palliative care might meet the needs of migrant patients and their families. Structural conditions including high staff ratio, more spacious accommodation, possibility of longer admissions, extended visiting hours. Personnel structures including greater emphasis on interdisciplinary teamwork, voluntary workers, greater availability of social workers and psychological support, greater clinical autonomy for nurses. Treatment intentions, different attitudes to EOL care, unconditional acceptance of subjective symptoms, focus on needs apart from illness, traditions/rituals etc., emphasis on EOL conversations, support for relatives. Identified staff characteristics, close relationships with patients, knowledge of rituals surrounding death and dying, willingness to ‘bend rules’, shield patients from overwhelming family members.	9/10 90%
Schrank et al., (2017) [53] Pushing boundaries-culture sensitive care in oncology and palliative care: a qualitative study. Austria	To understand how staff in multi professional health care teams in cancer care experience working with patients with different cultural backgrounds.	Qualitative study. Semi-structured interviews. 21 MDT members, medical, nursing, social work, psychology, spiritual care. 1 site providing cancer care, OPD, oncology day ward, oncology ward, palliative care ward and radiation oncology.	Culture-specific differences: large families, differing views on information sharing and EOL communication to general population, language barriers hindering communication, differing views on use of alternative treatment, more vocal expressions of emotion, little reporting of psychological symptoms, specific rituals/traditions around death. Reasons for differences: experience in home country/ socio-demographics/ isolation from wider Austrian society. Staff report sense of satisfaction and motivation when relationships are positive, also uncertainty and anxiety. Challenges include accommodating large families, communication difficulties due to language/interpreter issues, variable accommodations by staff causing tension. Tools for culture-sensitive care: organisational measures/ team-level measures/personal tools.	8/10 80%
Shahid et al., (2013) [54] Improving palliative care outcomes for Aboriginal Australians: service providers’ perspectives. Australia.	To explore care providers’ experiences and concerns in providing palliative care for Aboriginal people and to identify opportunities for overcoming gaps in understanding between them and Aboriginal patients and families.	Exploratory qualitative study. In-depth face-to-face or telephone interviews. 15 palliative healthcare providers. Hospital and home-based palliative care services.	Low uptake of palliative care services by Aboriginal people, lack of knowledge/understanding of palliative care and late referral are contributors. Historic mistrust of services, poverty, poor housing, remote locations with limited services influence engagement. Services offered not always acceptable to Aboriginal people. Challenges identified by providers include -communicating about death and dying -anger around death -family conflict over choices -family caregiving at EOL. Heterogeneity amongst Aboriginal communities and families noted. Discomfort /uncertainty accommodating rituals at end of life voiced by providers.	8/10 80%
Torres et al., (2016) [55] The ‘other’ in end-of-life care: providers’ understandings of patients with migrant backgrounds. Sweden.	To explore the understandings that end-of-life care providers articulate when they talk about challenges associated with cross-cultural interaction involving patients with migrant backgrounds.	Qualitative study. Focus groups. 60 MDT members, nursing, nursing assistants, medical, occupational therapy. 11 care settings, medicine/surgery/geriatrics/ specialised palliative home care/primary care.	Anticipating cross-cultural interaction to be challenging, assumption that ‘difference’ is challenging. Stereotypical views about migrant families but also about Swedish families. Expecting to encounter difficulty accommodating large families, concerns around physical space. Concerns about difficulty establishing relationships and communicating due to language barriers resulting in not being able to talk to patients about dying. Concerns around disclosure, Swedish laws. Respondents assumed ethno-cultural difference rendered patient-centred and culturally competent EOL care impossible.	9/10 90%
Vincent et al., (2019) [56] Provision of comprehensive culturally competent palliative care in the Qikiqtaaluk region of Nunavut. Canada	To explore health care providers perspectives on the provision of palliative care in the Qikiqtaaluk region of Nunavut, further examining their understanding of and experiences with providing palliative care services to Indigenous patients.	Qualitative exploratory study based on a constructivist paradigm. In-depth interviews, face-to-face or telephone. 7 physicians, 6 nurses. Small local acute care unit, community services.	Respecting Inuit culture, EOL planning and role of family. Recognising ‘sense of home’. Being aware of limited resources. Recognising role of medical interpreters. Improving quality of palliative care programme.	8/10 80%
Washington, K.T., Khosla, N. and Lero, C. (2019) [57] U.S. providers’ perceptions of the psychosocial needs of seriously ill patients of South Asian origin: implications for health social work. USA	To explore health care providers’ perceptions of the psychosocial needs of seriously ill patients of South Asian origin in the US.	Multi-method qualitative descriptive study. 4 focus groups, 35 participants, 23 individual interviews. 57 participants -nurses -physicians - physician assistant - nurse administrators – chaplains - medical social workers. 1 large university-owned health system, 1 large non-profit healthcare organisations, community-based hospices.	Patients faced financial and legal challenges; some immigration related. Perceived preference for family-based care and decision making, non-intrusion by care providers. Reluctance among family members to discuss emotional or psychological pain. Care providers attributed psychological problems to culturally incongruent care rather than patient or family related. Acculturation can cause differing opinions within families. Preference for support from within South Asian community rather that via health care system.	6/10 60%

### Quality assessment of the included studies

The Critical Appraisal Skills Programme (CASP) and its supporting user guide were utilized to assess the quality of the 26 included papers. CASP allowed the authors undertake a systematic and rigorous approach through a series of ten questions, prompting the authors to consider three areas when appraising qualitative studies, the validity of the results, the nature of the results and the contribution of the results [30]. The methodological quality of the papers assessed was generally good with CASP scores ranging from 50 to 100% (Supplementary file 2).

### Data analysis

Braun and Clarke’s six-step thematic analysis inductive approach [31] guided the data analysis process. At first, each paper was thoroughly read to highlight relevant quotes and paragraphs, with open notes made of initial ideas and concepts. Following this quotes and paragraphs with their notes were collated into an entire data set and preliminary open coding conducted to help streamline and converge the data. Here a color-coding system assisted in highlighting patterns across the data and immersion assisted with familiarisation of the data and patterns were noted, reflected upon and discussed. This process enabled the development of broader, more conceptualized themes and the reflection and discussion process allowed for the merging and/or refining and discarding some preliminary themes. Themes were then defined and labelled followed by critically reviewing each theme and coding to assess the accuracy of the coding process leading to verification of theme.

### Findings

Through data analysis four key themes emerged from the literature (communication and connection, the role of the family in death and dying, the role of education in addressing uncertainty and institutional and societal factors) and these are presented. In addition, the characteristics of the studies include in this review are presented in Table 3.

**Table 3:** Characteristics of the included studies

Number of included papers	26
Papers	Multiple papers reporting on different aspects of a single study (16 papers reported on 6 research studies). One study across 4 papers • Johnstone et al. [40, 41, 41, 42, 42, 43, 43, 44] • Washington et al. [33, 57] and Khosla et al. [44, 45, 45, 46, 46, 47] One study across 2 papers • de Graaff et al. [34, 35, 35, 36] • Roider-Schur et al. [52, 53] and Schrank et al. [53, 54] • Nielsen et al. [50, 51, 51, 52] • Torres et al. [55, 56] and Milberg et al. [48, 49]
Study design	Qualitative studies • Grounded theory - Bellamy and Gott [32], Green et al. [38, 39] • Ethnography - Nielsen et al. [50, 51, 51, 52] • Phenomenological – Debesay et al. [33], Henry and Timmins [39, 40] • Qualitative exploratory descriptive design - Johnstone et al. [40, 41, 41, 42, 42, 43, 43, 44], Shahid et al. [54, 55]and Vincent et al.[56, 57] • Qualitative approach not specified - de Graaff et al. [34, 35, 35, 36], de Voogd et al. [36, 37], Eckemoff et al. [37, 38], Khosla et al. [44–46], Mahilall et al. [47], Milberg et al. [48], Neiman [49], Roider-Schur et al. [52], Schrank et al. [53], Torres et al.53 and Washington et al. [57]
Country of origin	• Austria [52, 53] • Australia [38, 40–43] • Canada [50, 51, 56] • Ireland [39] • Netherlands [34–36] • New Zealand [30] • Norway [33] • Sweden [48, 55] • United States [37, 44–46, 49, 57] • South Africa [47].
Data collection methods	• Interviews, face-to-face – Bellamy and Gott [32], Debesay et al. [33], de Graaff et al. [34, 35], Eckemoff et al. [37], Henry and Timmins [39], Johnstone et al. [40–43], Khosla et al. [44–46], Neiman [49], Nielsen et al. [50, 51], Roider-Schur et al. [52], Schrank et al. [53], Shahid et al. [54], Vincent et al. [56], Washington et al. [57] • Telephone interviews - Shahid et al. [54], Vincent et al. [56] • Focus groups - Bellamy and Gott [30], de Voogd et al. [36], Khosla et al. [44–46], Green et al. [38], Mahilall et al. [47], Milberg et al. [48], Neiman [49], Torres et al. [55] and Washington et al. [57] • Observation - Nielsen et al. [50, 51]
Themes	• Communication and connection [33–48, 48, 49, 49, 50, 50, 51, 51–54, 54, 55, 55, 56, 56, 57, 57] • The role of the family in death and dying [33] • The role of education in addressing uncertainty [33, 34, 34, 35, 35, 36, 36, 37, 37, 38, 38, 39, 39, 40, 40, 41, 45, 46, 46, 47, 47, 48, 48, 49, 49–52, 52, 53, 53, 54, 54, 55, 55, 56, 56, 57, 57] • Institutional and societal factors [33–43, 43, 44, 44–46, 46, 47, 47, 48, 48, 49, 49, 50, 50, 51, 51, 52, 52, 53, 53, 54, 54, 55, 55, 56, 56, 57]

### Communication and connection

Managing care when the patient does not speak the primary language of the country featured in the majority of studies [3, 34–38, 40–43, 45–49, 51–53, 55–57] and was commonly identified as a barrier to the provision of comprehensive care. Practitioners voiced concern that usual methods of providing support or comfort were impaired [34, 36, 48]. Participants in two studies noted that the *snatches of conversations* [38] and *all of those small things that you say all the time* [48] that help to build relationships, were missing in the absence of a common language. de Voogd et al. [36] highlights the risk of misinterpretation when statements of wishes are taken literally and viewed through the care providers’ own standpoint without exploration or clarification.

Interpreters were seen as key to addressing communication issues however, difficulties accessing the services of interpreter and variability in quality are noted [33, 37, 38, 45, 46, 48–50, 52–56, 56, 57, 57]. Green et al. [38] noted that interpreters tend only to be requested for more formal discussions around issues such as consent. Difficulties also existed when using telephone interpreter services with inadequacies of a *relay conversation* [37], *difficulty with hearing* [49] and the missing of non-verbal cues [45, 46] evident. Practitioners reported using interpreters infrequently, citing the need for advance planning and reported that in general, families preferred to interpret for themselves [34, 37, 38, 45]. The use of family interpreters raised concerns regarding the burden it may place on family members and the extent of the information conveyed [34–38, 45, 48, 53, 55, 57]. A number of studies note that practitioners at times felt that families may modify the information they were trying to convey in an attempt to shield the patient [34–36, 38, 45, 53, 56]. The utilisation of staff members as interpreters raises some concerns due to the perceived burden [53]. There was evidence of positive efforts made by practitioners to ensure patients could be understood and this was seen as a way of establishing trust [38, 40, 42, 43]. To support understanding, practitioners sometimes used unorthodox methods such as drawing up word lists [36], drawing pictures [49], using sign language [34, 55] and using an app on practitioners’ personal phones [49]. Communication triads involving healthcare practitioners, family members and the patient were utilised with support strategies such as additional time spent establishing relationships with the family [34]. While practitioners rated communication to be either *moderate* or *good* [34, 35] concerns existed regarding the content of translated information relayed to the patient as patients sometimes did not understand a particular issue but are reluctant to seek clarification [49]. Communicating in the patients’ preferred language is both an ethical and licencing responsibility [37, 38].

The importance of making connections with patients and families to forge relationships is documented in all studies. A specific emphasis on presence is discussed in a number of studies [36, 39–42, 49, 52] expressed as *having a presence at the bedside, constantly checking yet giving space* [40, 41]. Value is placed on building and maintaining trust [38, 41–43] and demonstrating acceptance and concern by asking about preference and ascertaining usual ways of operating while respecting difference [34, 36, 38, 40–43, 49, 51–53, 56]. *Seeking and building on similarities is also thought to facilitate connection *[34, 38, 43, 53].* Intense expressions of distress and grief by family members were noted in a number of studies *[39, 48, 53, 54, 57]. *Practitioners emphasised the importance of providing physical space to facilitate such expression *[54, 57] *and emotional support for families at such times *[52]. *The importance of not labelling families also features *[42].

### The role of family in death and dying

All studies revealed the central role played by the families of culturally diverse patients in receipt of palliative care as perceived by practitioners. The large number of visitors was commented by practitioners working in in-patient settings [30, 38, 39, 45, 48, 49, 52, 53, 55]. Practitioners sought to support and educate families to fulfil patient and family wishes for participation in care and endeavoured to *work around *the family so that they would feel included [30]. Strategies to develop relationships with family members such as *actively involving family members…asking the family…listening to family *suggest that family involvement is largely significant in achieving a good death [40, 42]. However, ambivalence to family involvement is expressed by some practitioners, suggesting that they could not care for the patient because the family *want to care for their loved ones in a completely different way* [55]. Preparation of the family for death was important and involved explanations of what was happening and what might broadly be expected [38, 40–42, 50, 52, 56]. The pressure that care provision places on family members themselves was acknowledged by practitioners reporting a sense of duty in addition to family and community expectations [35, 36, 38, 54, 57]. However, a general reluctance to discuss psychological distress by family members themselves may exist and this can hinder practitioners fulfilling their caregiving role [57].

Tensions between practitioner values and family wishes occurred in many of the studies where issues arose concerning different models of decision making, non-disclosure and differing attitudes to patient autonomy [30, 33–38, 45, 46, 48, 51–56]. Devolved or collective decision-making was discomfiting for many practitioners and was frequently thought to result in withholding information from the patient [30, 34–38, 45, 47, 52, 53]. Difficulties also arose in identifying the family spokesperson/decision-maker [34, 35, 45, 46] and such decision-making was sometimes thought to impede female autonomy [53]. While acknowledging on-going professional concerns about decision-making and autonomy, care providers used a variety of approaches to address these concerns including adapting word choices, allowing additional time to hear families views and striving for an approach that is acceptable to patient, family and practitioner [34–38, 42, 43, 45, 53]. A further tension involved family preferences around the administration of medications, particularly opioids and practitioners perceived that patients suffered because of family members opposition to medication use [36, 38, 39, 49]. Furthermore, when a trusting relationship had not been established, families and patients were more likely to reject medications [43] and fear of addiction, side -effects, stoicism and lack of understanding arising from attitudes to analgesia in country of origin were also identified [44].

There was a perception that speaking about death was disliked by patients and families from culturally diverse backgrounds [32, 33, 35, 38, 43, 45, 51–56]. Practitioners often held a view that death is a taboo topic that people did not want to talk about [33, 51, 54] and that patients were reluctant to die at home due to cultural influences [51]. However, practitioners also noted, within the same studies, that some families were very open to discussing impending death [51, 54] and that reluctance to die at home was possibly due to lack of knowledge of available supports and fear of burdening family members [51, 52]. A particular difficulty arose for practitioners when the parents of young children were involved and were reluctant to talk about death, here practitioners reported feeling complicit in *concealing the truth *and had considerable concern for the families post bereavement [33, 34]. The significance of rituals and practices around death and the resultant implications for practice were evident [38–40, 45, 47, 49, 52–54, 57] with efforts made to facilitate families *they have very strong rituals and beliefs* [49]. Practitioners expressed this willingness as *doing the right thing* [40] and expressed satisfaction with been able to support cultural practices where they were *walking alongside *patients and families [38]. Support varied from family to family highlighting an individualised approach [32, 38, 39, 47, 54] and there was a readiness to be flexible to accommodate family wishes and rituals which often required *bending the rules* [49, 52, 53]. Practitioners observed that although culture had a bearing on care, certain aspects of the approach of end of life were common to all people irrespective of culture, ethnicity or religion [32, 38, 43, 51] and cultural considerations were integrated into culturally responsive individualised patient care [32, 34–36, 38–43, 49, 51–53, 56].

### The role of education in addressing uncertainty

All the studies documented challenges encountered by practitioners when providing palliative care to culturally diverse patients and their families, with uncertainty a common thread. Feelings of *inexperience, unease, and helplessness* [39, 40], *overwhelmed and underprepared* [40, 41], *anxiety, helplessness, self-insufficiency and uncertainty* [53, 54], and *uncertainty, stress, frustration* [48, 49] were expressed. Practitioners noted that they were more familiar with practices, communication cues and family dynamics in native born patients and families [33, 34, 38, 39, 48, 55] and were concerned about causing offence or upset by contravening unfamiliar customs [33, 34, 39, 40, 40, 41, 48, 48–50, 50]. These concerns resulted in practitioners questioning the quality of care they provided [33, 34, 36, 45, 46, 48, 54, 55] as family members views about care were sometimes at variance with their own professional ideals and values [33, 45, 46, 48, 55]. On occasions, practitioners stepped back from patients due to discomfort at the unfamiliarity of certain situations [48, 49]. Practitioners attempted to overcome uncertainties by a constant adjustment to reconcile differences in approaches [51], proceeding slowly and with care in partnership with the patient and family [38–43] .Other practitioners sought understanding and joint solutions while acknowledging conflicting opinions and on occasions, resolution did not occur [34, 35]. Where practitioners provided culturally appropriate care they reported satisfaction [38, 53] and found support and learning from colleague’s experiences [40].

A lack of knowledge or a need for further education was voiced by practitioners [33, 39, 40, 48, 49, 54–56]. Some nurses had requested training and education [33, 56] due to the changing landscape of care *we are coming across more people from abroad, different culture, it’s time we knew, we need to know.* [39] Healthcare practitioners felt that the lack of knowledge could hamper their ability to care for culturally diverse patients but that more knowledge about that which was less familiar would make cross-cultural encounters feel less *unusual* [33, 48, 55]. Few studies document previous education in cultural diversity. In one, there is evidence of a yearly *class on cultural competence* [37] and in another, only seven out of twenty participants had formal education in the area [40]. Practitioners in this study [40], acknowledged a lack of knowledge and understanding *I was ignorant *and purposefully sought to increase their knowledge informally through questioning and observing more experienced colleagues and engaging with family members. In addition, attending seminars and learning from interdisciplinary discussions were deemed helpful [47, 53].

### Institutional and societal factors

The influence of factors rooted in the wider context of palliative care provision to culturally diverse patients occur in all the studies reviewed. There is a perception of low levels of awareness of and engagement with palliative care services amongst patients and families [32, 37, 46, 48, 49, 51, 53, 54]. Reasons for this are manifold, including late referrals to palliative care services [54],a perception of hospice as *white and middle class* [32], unfamiliarity with the concept of palliative care [34, 36, 37, 42, 44, 46, 51, 54] and a lack of familiarity with health and palliative care services [35, 37, 44, 46, 48, 49, 53–55]. Influences on care such as social isolation, economic disadvantage, experience of discrimination due to religion or other factors, having endured war or trauma, level of education, and length of time in host country were also perceived to influence interaction [35, 38, 46, 54]. Specific support, such as financial information, spiritual or bereavement support provided by the community from which the patient originates can play an important role in supporting engagement with palliative care services [36, 37, 39, 44, 46, 57].

Several factors pertaining to healthcare institutions, specifically related to resources occur throughout the studies. The provision of adequate pastoral care services was seen as particularly helpful [38, 39, 47, 53, 57]. For indigenous patients and families, remote geographical location had a significant impact on the provision of palliative care with limited infrastructure and personnel available [54, 56]. Environmental factors such as the importance of the physical environment to accommodate large family groups and facilitating gender preferences were seen as important aspects of care [32, 36, 45, 49, 52, 54, 55, 57]. However, it was not always possible to facilitate and while attempts were made to adjust rosters to try and meet patient preferences for carers of a particular gender, it could not be always facilitated/accommodated as *we don’t have a list, sometimes there are only men on the night shift.* [36]

The experience of a culturally diverse workforce is an important factor but assigning nurses to patients of a similar cultural background did not always result in language congruence and could result in stereotyping [51] and even with a shared migrant background, issues of unfamiliarity were still encountered [40]. In addition, practitioners reported challenges when dealing with patients and families from a similar cultural background, where increased workload [51] and unease when conflicts occurred were noted [45, 46, 53]. Practitioners reported sense of satisfaction at being able to assist those of a similar background [40, 43, 53] and diversity in personnel was viewed as a positive means of meeting patients’ needs [36, 40]. The management of issues which arise when caring for culturally diverse patients and families at ward level should be discussed as a team and efforts made to come to a consensus on approaches used [47, 53]. It is suggested that endeavours to accommodate patient preferences, such as extending visiting times etc., can be a source of disagreement within a team so ongoing dialogue, discussion and team support are warranted [53, 54].

## Discussion

This systematic review explored the perspectives of healthcare practitioners on providing palliative care to patients with culturally diverse backgrounds in a variety of settings. Findings concur in some respects with van Eechoud et al’s. [27] systematic review of oncology health workers caring for ethnic minority patients. Of similarity is the principal challenges of communication and a fear of acting in a culturally insensitive way but extending on this the findings from this review highlights the pivotal role of the family, the need for an individualised approach to care, the universality of needs when approaching end of life and the need for education as voiced by practitioners. Challenges posed to healthcare practitioners by lack of a common language with patients were found in this review [32, 34–38, 40, 41, 45–49, 51–53, 55–57] and this is reflected in the wider literature concerning interactions between culturally diverse patients and healthcare practitioners in palliative care contexts [15, 58, 59]. Difficulty accessing professional interpreters, a reluctance to use them by staff, by family or patient or dissatisfaction with the service provided, suggests a myriad of factors influencing their use [34, 38, 45, 48, 49, 52, 53, 55, 56, 56, 57]. Nonetheless engagement with professional interpreters has been shown to aid in reducing healthcare disparities by reducing errors and through improved communication, leading to greater patient satisfaction [18] and demonstrates clinicians’ respect for patients [60]. Research identifies that interpreters are frequently not utilized even when practitioners are aware that their use is advisable with family and bilingual staff utilized instead [61, 62]. However, such an ad hoc means of translation is inadvisable and indicates that defaulting to use of family without exploration of alternatives or family and patient opinions does not best serve the interests of healthcare practitioners, families, or patients [63, 64].

A clear finding of the review is the means by which practitioners built relationships with patients and families [32, 36, 38–43, 49, 52, 53]. and it is recognised that intentional presence can convey compassion in intercultural encounters [65]. Culturally diverse patients report feelings of respect and support when practitioners spent time with them and try to communicate even in the absence of a common language [66] with a sense of exclusion reported in the absence of such efforts [60]. The need for time as an aid to communication and relationship building is acknowledged elsewhere [67]. Consistent with existing research this review also highlights the importance of family in the care of culturally diverse patients in receipt of palliative care [68, 69]. The importance of a physical environment which supports large families was noted in several studies [32, 52, 57] and is also seen in the literature as an important asset to inpatient care and helpful to both patients, families, and practitioners [70, 71]. While healthcare practitioners perceived that culturally diverse patients generally enjoyed good support from immediate and extended families, concerns are evident in one study regarding the difficulties family members have accepting responsibility for care of a patient at home [54]. Practitioners demonstrated awareness that particular aspects of patients and families migration history may have a bearing on care provision [34, 35, 37, 38, 42, 44–46, 49, 53, 57] and recognised the demand placed on female family carers [35, 38]. This burden is recognized in the wider research [72, 73] which also notes that a sense of responsibility for older family members in people with culturally diverse backgrounds does not automatically mean close emotional bonds [74, 75]. This is an important factor as support for family members is an integral part of palliative care [75] and that the presence family does not negate the pressures that the caring role may place on some family members of migrant and indigenous families [71–73].

An expected finding from the review which occurred within all studies, is the complexity involved for practitioners in negotiating with the patient and family around such matters as perceived cultural taboos. The review reveals that widely held views about death as a taboo subject in certain cultural groups is not consistently realised [42, 50, 51, 54]. This variability is echoed in the wider literature [76–78] further illustrating the need for individualized assessment and the need for practitioners to be aware that cultural taboos are a reality for some but that subscription to them is not uniform and individual consideration is necessary as other influences such as relationships, fear, lack of awareness of services may be relevant. A notable finding from the review is concern for patients’ autonomy where family play a greater role in decision making and disclosure [32, 33–39, 45, 46, 48, 49, 52, 53, 55, 57] and this resonates with research in oncology and palliative care where devolved decision making and a preference for non-disclosure are noted [79, 80]. Language barriers and the difficulties ascertaining patient preferences are found to make information sharing a complex matter [27, 81]. Thus practitioners adjust communication as each situation is assessed [36–38, 40–43, 45, 46, 49, 51–53] and particularly when home visiting is involved [34, 35, 39, 54, 57]. The desirability of ascertaining early from both patient and family, what their preferences are regarding information sharing and documenting their preferences is essential for working together [60].

Ascertaining the role of culture in the lives of patients, families, and clinicians themselves and negotiating and adapting care necessitates a nuanced and informed approach. Practitioners demonstrated an awareness of culture as a dynamic, evolving process i.e. that patients and families may embrace aspects of culture to varying degrees and specifically spoke of the need to avoid generalizations [32, 38, 40, 43, 47, 51]. Reliance on a patients’ cultural background and beliefs alone can result in cultural stereotyping [21] and individualised approaches were frequently utilised as a means to overcome this risk [32, 33–43, 45–47, 51–54] an approach supported by the wider literature [26, 63]. However, individualised care must be delivered with awareness of the myriad of potential influences on the intercultural encounter i.e. cultural, socioeconomic, educational and family relationships and where culture is seen as part of a patients’ *complex personhood *rather than a separate entity [82]. The findings of this review demonstrate that the ways care is adapted to try to meet the varying needs of patients and families are similar with all people irrespective of cultural background [32, 34, 35, 38–43, 45, 49, 51, 52], indicating that practitioners draw on skills such as sensitive communication, individualised care and family support which are integral to the practice of palliative care [83] and therefore familiar to practitioners. This suggests that the patient-centred practice of palliative care can provide a basis for incorporating cultural considerations into every encounter by acknowledging the universality of differing communication styles, need for information, family involvement and health beliefs and negotiating these with an awareness of of how culture interconnects with these [84, 85].

Generally, practitioners experienced uncertainty when encountering patients with an unfamiliar cultural background and this is reflected in the wider literature [18, 58, 59]. Within a systematic review of barriers to care of ethnic minority patients, nurses report communication difficulties, lack of culturally appropriate resources and insufficient education were primary concerns [86]. Practitioner reported knowledge deficits and requests for further education are evident in this review [33, 39, 40, 48, 49, 54–56] and acknowledged within the wider literature [19, 58, 87]. Although further education is seen as a means to improve the care offered and to help allay uncertainties [33, 39, 48, 57] culture specific knowledge is recognised as potentially contributing to stereotyping and encouraging generalisations [26, 84] and the literature does not offer a simple solution. Studies examining cultural competence education, report that there is little assessment of outcome [88] and are seldom evidence based [89]. *Instead,it is suggested that enhancing communication skills and expanding awareness of the many factors, clinician, patient and family related, which influence communication may prove beneficial.* [89]. A systematic review assessing the effectiveness of cultural competence on patient satisfaction found that while training improved patient satisfaction, concerns existed regarding the quality and methodological rigor of the reviewed studies, so caution was advised on interpretation of results [90]. Nonetheless, perceived gaps in knowledge contribute to a sense of uncertainty about aspects of care provision, thus within educational settings, teaching students to approach situations which are unfamiliar, with interest rather than unease is pivotal [91]. Education in intercultural communication and an ability to question how care is provided are required to equip future clinicians to provide appropriate care for all [91]. Within training programmes there needs to be an integration and assessment of culturally sensitive care throughout curricula [92, 93] and a combination of approaches is required to prepare nursing students to provide care to culturally diverse patients, including knowledge, simulation of cross-cultural encounters, reflection, and appropriate role models [94]. For existing practitioners, the training of staff members to become ethnic coordinators had a beneficial influence on patient related interactions and on the culture of care towards culturally diverse patients [95]. A combination of a broad knowledge of culture relating to health and to death and dying, enhanced communication skills development and self-reflection can offer practitioners a framework from which to approach clinical encounters [96]. It is also recognised that educating practitioners to use moral reasoning could help promote ethical and just decision making when exploring care for patients with culturally diverse backgrounds [97, 98].

While there is awareness amongst practitioners in all studies of the institutional and societal factors which may influence the delivery of care, the wider literatures evidences low levels of engagement with palliative care services among some culturally diverse patients [22, 25]. Palliative care is unfamiliar to some culturally diverse patients as the term does not translate directly in many languages [82]. A systematic review of referrals to palliative care from oncology/haematology services identified a lack of knowledge of palliative care amongst healthcare practitioners and low levels of awareness of palliative care amongst patients and families in the general population, is a significant and widespread obstacle to palliative care referrals [99]. It has been highlighted that gatekeeping by referrers who perceive that culturally diverse patients do not utilise palliative care services can occur [100] but it has also been shown that palliative care referrals were proportionally higher in hospital settings for culturally diverse patients and posited that more opportunity and time to explore understanding may have been a contributing factor [101]. The provision of time is of critical importance in the provision of patient-centred care during the palliative phase of illness and the ability to spend time with patients and families is vital to building trusting relationships [83, 102]. Limited time resources are recognised as a barrier to care for culturally diverse patients [103]. This highlights the role of organisational support in facilitating practitioners to have time as a resource [33, 47, 52, 54]. Furthermore, the existence of a culturally diverse workforce in healthcare settings is viewed as a facilitator of good care [14, 22, 104] but may place additional demands on culturally diverse practitioners which may impact the practitioners themselves [105].

### Strengths and limitations

A strength of this review is the insight gained into the perspectives of healthcare practitioners providing palliative care to patients with a culturally diverse background. However, it is recognised that the time frame may have eliminated papers that were relevant and could have added to the current body of literature. This review included papers that were published in English, however given the subject topic of culturally diverse backgrounds the review may have eliminated papers of importance as there were not written in English. This review highlights the difficulties for healthcare practitioners caring for patients and their families from diverse cultural background and suggested strategies that could help meet the needs of their patients. Research into this topic is limited and so encompassed all multidisciplinary members and it is possible that had the review been restricted to a single professional group, the findings may be more transferable. However, given the review explored healthcare practitioner’s perspectives of providing palliative care to patients from a culturally diverse backgrounds all practitioners were included, and findings may be transferable.

## Conclusion

In this review, it is apparent that healthcare practitioners face challenges providing palliative care to patients from a culturally diverse background. Language barriers, divergent views on autonomy, uncertainty about aspects of culture and a perception of a need for further education are all issues practitioners dealt with in practice. However, practitioners draw on their existing skills to adapt their practice to unfamiliar situations and utilise palliative care’s patient centred approach to ascertain needs and involve the family. This approach allows them to be aware of the influences that belonging to an indigenous or migrant community may bring but greater integration of culture needs to be incorporated within undergraduate and post graduate education. Ethnicity is not currently recorded in some countries for example on the Cancer Registry in Ireland therefore to help inform and develop services on a national level ethnicity should be included.

### Supplementary Information

**Additional file 1: Supplementary file 1.** PRISMA CHECKLIST. **Supplementary file 2.** Quality appraisal - CASP Appraisal scores

## Data Availability

Data used for analysis in this review are all extracted from the original published reviews and are presented in Table 2 (Data extraction table).
